# Can Short-Term Ocular Dominance Plasticity Provide a General Index to Visual Plasticity to Personalize Treatment in Amblyopia?

**DOI:** 10.3389/fnins.2020.00625

**Published:** 2020-06-26

**Authors:** Chunwen Tao, Zhifen He, Yiya Chen, Jiawei Zhou, Robert F. Hess

**Affiliations:** ^1^State Key Laboratory of Ophthalmology, Optometry and Vision Science, School of Ophthalmology and Optometry and Eye Hospital, Wenzhou Medical University, Wenzhou, China; ^2^Department of Ophthalmology and Visual Sciences, McGill Vision Research, McGill University, Montreal, QC, Canada

**Keywords:** amblyopia, interocular suppression, ocular dominance plasticity, visual acuity, patching therapy

## Abstract

**Purpose:**

Recently, Lunghi et al. (2016) showed that amblyopic eye’s visual acuity *per se* after 2 months of occlusion therapy could be predicted by a homeostatic plasticity, that is, the temporary shift of perceptual eye dominance observed after a 2-h monocular deprivation, in children with anisometropic amblyopia. In this study, we assess whether the visual acuity improvement of the amblyopic eye measured after 2 months of occlusion therapy could be predicted by this plasticity.

**Methods:**

Seven children (6.86 ± 1.46 years old; SD) with anisometropic amblyopia participated in this study. All patients were newly diagnosed and had no treatment history before participating in our study. They finished 2 months of refractive adaptation and then received a 4-h daily fellow eye patching therapy with an opaque patch for a 2-month period. Best-corrected visual acuity of the amblyopic eye was measured before and after the patching therapy. The homeostatic plasticity was assessed by measuring the temporary shift of perceptual eye dominance from 2-h occlusion of the amblyopic eye before treatment. A binocular phase combination paradigm was used for this study.

**Results:**

We found that there was no significant correlation between the temporary shift of perceptual eye dominance observed after 2-h occlusion of the amblyopic eye and the improvement in visual acuity in the amblyopic eye from 2 months of classical patching therapy. This result, although in disagreements with the conclusions of Lunghi et al. involving the short-term patching of the amblyopic eye, is in fact consistent with a reanalysis of Lunghi and colleagues’ data.

**Conclusion:**

The short-term changes in perceptual eye dominance as a result of short-term monocular deprivation do not provide an index of cortical plasticity in the general sense such that they are able to predict acuity outcomes from longer-term classical patching.

## Introduction

There is a considerable variability in the response to amblyopic treatment, be it classical occlusion therapy (Stewart et al., 2005) or binocular therapy (Hess and Thompson, 2015) across a population of amblyopes of all ages. Unfortunately, there is no way of knowing which patients are more likely to respond to a particular treatment prior to therapy. Usually, it is not until the end that the patients who respond to treatment can be separated from those who do not. Not all of this variability can be attributed to the difference in compliance (Stewart et al., 2005, 2007; Fronius et al., 2014), leading us to the inescapable hypothesis that some amblyopes have brains that are more capable of change, in other words, more plastic, than others. If this is the case, we might be able to assess some general measures of visual cortical plasticity to better personalize the present treatment.

Recent studies suggest that short-term visual deprivation of one eye temporally modulates the perceptual eye dominance of normal adults (Lunghi et al., 2011). Comparable effects occur in adults with amblyopia (Zhou et al., 2013c). This new form of plasticity has been shown in both binocular rivalry and binocular combination measures (Lunghi et al., 2011, 2013; Zhou et al., 2013a, c, 2014, 2017a,b; Lunghi and Sale, 2015). There is evidence that it involves a reciprocal change in the sensitivity of each eye’s input; the previously patched eye becomes more dominant, and the previously unpatched eye less dominant, that is, a homeostatic form of plasticity (Zhou et al., 2013a; Chadnova et al., 2017). It is therefore the opposite to what occurs in early critical period plasticity as a result of long-term monocular deprivation. The underlying mechanisms of short-term patching-induced perceptual eye dominance plasticity (for short, we use the term of “perceptual eye dominance plasticity” in this article) is not fully understood. Electrophysiology (Lunghi et al., 2015a; Zhou et al., 2015) and brain imaging (Lunghi et al., 2015b; Chadnova et al., 2017; Binda et al., 2018) studies suggest that the early visual cortex is involved. For example, using steady-state visual evoked potential (SSVEP), Zhou et al. (2015) found reciprocally shifted responses to the two eyes’ visual inputs in the primary visual cortex; similar results were also found in an magnetoencephalogram (MEG) study (Chadnova et al., 2017). Chadnova et al. (2017) postulated that patching modulates the contralateral inhibition prior to binocular combination. Using functional magnetic resonance spectroscopy (fMRS), Lunghi et al. (2015b) showed that the short-term monocular deprivation–induced perceptual eye dominance shifts were linked to reduced levels of γ-aminobutyric acid in the primary visual cortex. A recent functional magnetic resonance imaging study (Binda et al., 2018) also suggests that it involves the primary visual cortex in this form of plasticity.

Short-term monocular patching–induced perceptual eye dominance shift represents a measure of visual cortical plasticity. It could potentially be a general index to how modifiable the visual areas of the brain are. Unlike training-induced visual plasticity (Zhou et al., 2006; Huang et al., 2008), perceptual eye dominance plasticity occurs after a short period (0.5–5 h) of monocular patching (Min et al., 2018, 2019). It therefore might be an efficient means to personalize treatment for amblyopia if there was a strong correlation between the magnitude of perceptual eye dominance plasticity and the benefits from long-term treatment. By using a binocular rivalry paradigm, Lunghi et al. (2016) observed that children who exhibit a higher degree of short-term perceptual eye dominance plasticity (determined after a 2-h period of patching session) exhibit a larger “recovery rate” of the amblyopic eye after long-term (months) patching procedures; the “recovery rate” is defined as the absolute final visual acuity after the long-term patching. According to Amblyopia PPP (Wallace et al., 2018), the success of patching therapy only makes sense if it is measured incrementally, that is, the “improvement in visual acuity.” Therefore, whether changes in perceptual eye dominance plasticity are able to predict *improvements* (i.e., difference between initial acuity before occlusion therapy and that found after occlusion therapy) in visual acuity (i.e., the effects of patching therapy) as a result of long-term patching is unclear.

We directly tested this idea in this study. Initially, we measured perceptual eye dominance plasticity by patching the amblyopic eye for 2 h. Subsequently, classical occlusion therapy with an opaque patch occluding the fellow eye (4 h/day for 2 months) was undertaken in seven newly diagnosed patients. Any patient who needed an update to the spectacle correction had been allowed a 2-month period before undertaking the experiment.

## Materials and Methods

### Participants

Seven children (6.86 ± 1.46 years old; SD) who had anisometropic or ametropic amblyopia and were able to perform the binocular phase combination task after practice participated in this study. All patients had been newly diagnosed and had no treatment history before they participated in our study. The clinical details of the patients and their visual acuity before and after 2 months of treatment are provided in Table 1. All participants were naive to the purpose of the study. Written informed consent was obtained from their parents or guardians before the start of the experiment. This study followed the tenets of the Declaration of Helsinki and was approved by the Ethics Committee of Wenzhou Medical University and McGill University.

**TABLE 1:** Visual acuity before and after 2 months of treatment.

ID	Age (years)	Cycloplegic refractive errors (OD/OS)	Visual acuity (logMAR)				Balance point (FE/AE) before treatment
			
			Before treatment		2 Months of treatment		
				
			OD	OS	OD	OS	
S1	5	−1.00/−1.00 × 180	0.275	0.575	0.175	0.375	0.432
		−6.00/−2.00 × 180					
S2	6	+1.50	–0.025	0.575	–0.025	0.275	0.285
		+5.00					
S3	8	Plano	–0.025	0.575	–0.025	0.475	0.192
		+2.50/+1.75 × 80					
S4	6	+3.50	0.175	0.675	0.175	0.275	0.158
		+4.00/+0.75 × 95					
S5	8	+4.50	0.475	–0.025	0.275	–0.025	0.329
		Plano					
S6	9	+4.00	0.575	–0.125	0.4	–0.125	0.135
		Plano					
S7	6	Plano	–0.025	0.875	–0.025	0.775	0.234
		+2.00/+1.75 × 85					

### Apparatus

The stimuli for the short-term monocular deprivation measurement were generated and controlled by a PC computer running MATLAB (MathWorks, Natick, MA, United States) with PsychToolBox 3.0.9 extension (Brainard, 1997; Pelli, 1997). The stimuli were presented on a gamma-corrected LG D2342PY 3D LED screen (LG Life Sciences, Seoul, South Korea) with a 1,920 × 1,080 resolution and a 60-Hz refresh rate. Subjects viewed the display dichoptically with polarized glasses in a dimly lit room at a viewing distance of 136 cm. The background luminance was 46.2 cd/m^2^ on the screen and 18.8 cd/m^2^ through the polarized glasses. Patients’ best-corrected visual acuity was measured monocularly using the Logarithmic Tumbling E Chart (Mou, 1966) at 5 m.

### Design

**FIGURE 1:**
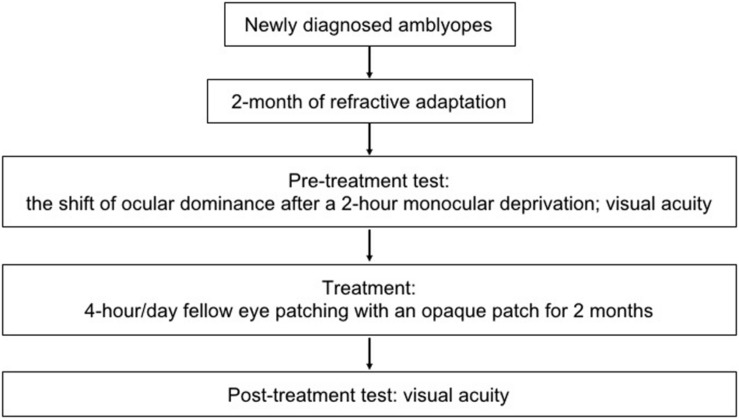
An illustration of the experimental design. Seven newly diagnosed child amblyopes (6.86 ± 1.46 years old; SD) participated, in which the treatment effect of 2 months’ patching therapy (4-h daily patching with an opaque patch) was tested after 2 months of refractive adaptation. The short-term monocular deprivation effect was quantified by the shift of perceptual eye dominance in binocular phase combination after 2 h of amblyopic eye patching before the initiation of the treatment.

In this study, the treatment effect of 2 months’ patching therapy (4-h daily fellow eye patching with an opaque patch) was tested after 2 months of refractive adaptation. The short-term monocular deprivation effect was quantified in an initial experiment by the shift of perceptual eye dominance in binocular phase combination after 2 h of amblyopic eye patching (Zhou et al., 2013c). An illustration of the experimental design is provided in Figure 1.

### Procedure and Stimuli

**FIGURE 2:**
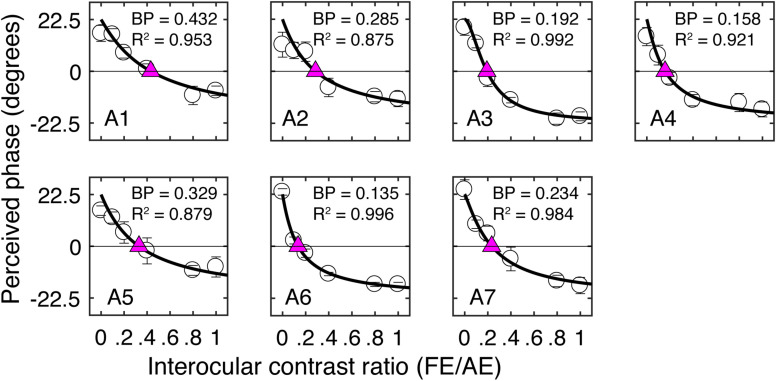
Individual’s binocularly perceived phase as a function of the interocular contrast ratio measured before the patching treatment. Each panel plots results of one patient. Error bars represent standard errors from eight repetitions of the test. The curve in each panel represents fits with a contrast-gain-control model (Zhou et al., 2013b). The purple triangle represents where the two eyes were balanced. The corresponding interocular contrast ratio (in short “BP”) and the goodness-of-fit are provided in each panel.

Similar to our previous studies (Zhou et al., 2013a), the short-term monocular deprivation effect was tested with a binocular phase combination task. In the measure, two horizontal sine-wave gratings (1 cycle/°, 2° × 2°), with equal and opposite phase shifts (+22.5° and −22.5°) relative to the center of the screen, were dichoptically presented to the two eyes. The perceived phase of fused stimuli was 0° when the two eyes contributed equally to binocular fusion. The interocular contrast ratio at that condition was the balance point in binocular phase combination. We first tested this balance point for each patient with the contrast of the stimuli in the amblyopic eye set as 100%. This was achieved by measuring individual’s binocularly perceived phase at interocular contrast ratios of 0, 0.1, 0.2, 0.4, 0.8, and 1, and the binocularly perceived phase versus interocular contrast ratio (PvR) curve was fitted with a contrast-gain control model (Ding and Sperling, 2006; Zhou et al., 2013b). One to 3 h of practice trials were provided before we conducted the main study to make sure patients understood the task and had a reliable performance in the binocular phase combination task. Individuals’ PvR curves measured before treatment are provided in Figure 2. The balance points of patients before treatment are provided in Table 1.

We then fixed the contrast of the stimuli in the two eyes based on the individual’s balance point and tested individuals’ binocularly perceived phase before and after a 2 h of monocular deprivation of the amblyopic eye with an opaque patch. Two stimulus configurations were used for measuring the binocularly perceived phase to account for positional bias: +22.5° phase in the amblyopic eye and −22.5° phase in the fellow eye, and −22.5° phase in the amblyopic eye and +22.5° phase in the fellow eye. The half of the difference between these two configurations was calculated as the binocularly perceived phase. Each session of binocularly perceived phase measurement contains 16 trials (two configurations × eight repetitions). The two configurations were randomly assigned in different trials. In each trial, observers were asked to adjust the position of a flanking reference line to locate the middle of the dark strip of the binocularly perceived grating to indicate its phase. A high-contrast frame (0.11° in width and 6° in length) with four white diagonal lines (0.11° in width and 2.83° in length) was continually presented surrounding the grating in each eye to help observers maintain fusion. Subjects normally needed around 3 min to finish one measurement session. We tested three sessions of binocularly perceived phase within 10 min after patients finished the 2 h of monocular deprivation. We averaged the results of these three sessions and then calculated the difference between the average post-patching perceived phase and the baseline to get the perceptual eye dominance difference index after the 2 h of monocular deprivation. We also normalized individuals’ perceptual eye dominance difference index to the largest one in this group. This normalization ensured that the normalized perceptual eye dominance difference index ranged from −0.5 to 1, similar to the range reported in the study of Lunghi et al. (2016). The normalization itself would not change any correlation analysis we conducted in this study.

For the best-corrected visual acuity measure, we asked patients to read the optotypes one after another and stopped when they could not respond within 10 s. We calculated their percentage correct at different lines of the logarithmic visual acuity chart. We then used linear interpolation to calculate the score associated with 75% correct judgments. This score was defined as patients’ visual acuity.

## Results

Figure 2 illustrates individual’s binocularly perceived phase as a function of the interocular contrast ratio measured before the treatment. The contrast-gain control model fit well to the data, with an average goodness-of-fit of 0.941 ± 0.054 (mean ± SD). This is similar to our previous observation (Zhou et al., 2013b) in adults with amblyopia (0.951 ± 0.022), indicating that our patients in this study were able to make reliable measurements with the binocular phase combination task before the treatment. Also, similar to our previous observation (Zhou et al., 2013b), there was a trend toward decreasing contrast ratios being associated with increasing interocular visual acuity differences (*r* = −0.59, *P* = 0.16).

**FIGURE 3:**
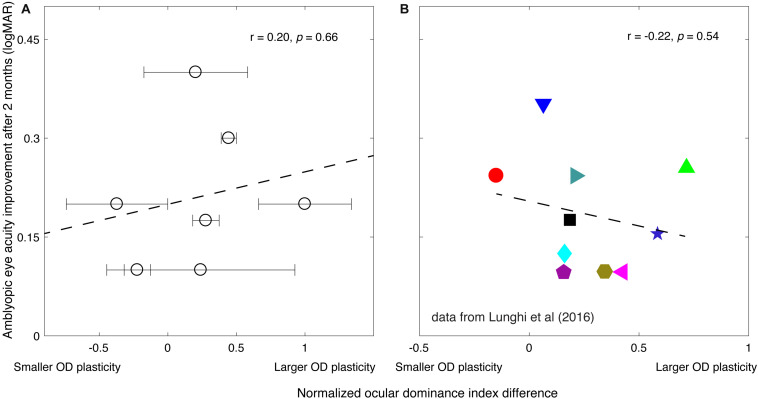
The relationship between the amblyopic eye acuity improvement after 2 months of treatment and the short-term monocular deprivation effect. **(A)** Results from the present study. The horizontal axis represents the effect of short-term monocular deprivation on the perceptual eye dominance (PED) shift. The value, if larger than 0, indicates the patched eye was getting stronger after the short-term monocular deprivation. The larger the value indicates the larger PED plasticity. The dashed line is a linear fit of the data. The error bars represent standard deviation based on the three posttest sessions measured within 10 min after patients finished the 2 h of monocular deprivation. A 2-tailed Pearson correlation analysis showed that the correlation between the amblyopic eye acuity improvement after 2 months of treatment and the normalized PED index difference was not significant: *r* = 0.20, *P* = 0.66. **(B)** Reports from Lunghi et al. (2016) study. Their work is licensed under a Creative Commons Attribution-Non-Commercial-NoDerivatives 4.0 International License. The results from Figure 4B and Table 1 in Lunghi et al. (2016) article are replotted here. Ten patients [6.2 ± 1.0 years (SD)] accepted Bangerter filter patching therapy (whole walking time in each day) after 2 months of refractive adaptation. The PED index difference was quantified using a binocular rivalry task before the start of the patching therapy. The dashed line is a linear fit of the data. A two-tailed Pearson correlation analysis showed that the correlation between the amblyopic eye acuity improvement after 2 months of treatment and the PED index difference was not significant: *r* = −0.22, *P* = 0.54.

Long-term patching therapy (4 h/day for 2 months) significantly improved the visual acuity of the amblyopic eye in our patients, from an average of 0.62 ± 0.05 to 0.41 ± 0.07 (logMAR): *Z* = −2.38, *P* = 0.018, 2-tailed Wilcoxon signed ranks test. In Figure 3A, we plot the amblyopic eye acuity improvement after 2 months of treatment as a function of the normalized short-term perceptual eye dominance index difference for the seven patients in our study. The normalized perceptual eye dominance index difference was larger than 0 in five of the seven patients, which indicates a strengthening of the patched eye after the 2-h short-term monocular deprivation. Two of the seven patients had a shift of perceptual eye dominance in the reversal direction. This pattern of result was similar to that previously reported by Lunghi et al. (2016), in which one of their 10 patients had a shift of perceptual eye dominance in the reversal way (Figure 3B). A two-tailed Pearson correlation analysis showed that the correlation between the amblyopic eye acuity improvement after 2 months of treatment and the normalized perceptual eye dominance index difference after 2 h of amblyopic patching was not significant: *r* = 0.20, *P* = 0.66.

## Discussion

We show that there was no significant correlation between the amblyopic eye’s acuity improvement after 2 months of occlusion treatment and the normalized perceptual eye dominance index difference associated with the perceptual eye dominance plasticity after 2-h occlusion of the amblyopic eye.

Our results seem to be in conflict with the claim made by Lunghi et al. (2016) that the change in perceptual eye dominance measured after 2-h of occlusion therapy predicts “the recovery rate” of the amblyopic eye in anisometropic children. However, Lunghi et al. (2016) in their original article plotted the change in perceptual eye dominance against the absolute acuity of the amblyopic eye at end of treatment. This is because they defined the effects of occlusion treatment (the “recovery rate”) in terms of the absolute visual acuity of the amblyopic eye measured after 2 months of treatment. This is an incorrect metric for the “recovery rate.” Their conclusion is not interesting because clinically, in patients with amblyopia, their final visual acuity after treatment is best predicted by their initial visual acuity before treatment. This is also true in Lunghi and colleagues’ study. According to Table 1 in Lunghi and colleagues’ article, there is a strong correlation between the amblyopic eye’s visual acuity before and after 2 months of treatment: *r* = 0.731, *P* = 0.016. The *p*-value is even smaller than that reported in Lunghi and colleagues’ article using the homeostatic plasticity (ρ = −0.65, *P* = 0.04). This suggests that if one’s objective were to predict amblyopic eye’s absolute visual acuity after 2 months of occlusion therapy, one could simply rely on patients’ initial visual acuity rather than a complicated psychophysics measure. What one really would want to do is to predict what change would occur in acuity as a result of treatment. That is what we set out to do.

Thus, our purpose was to investigate the effects of the occlusion therapy, in terms of the visual acuity improvement (i.e., the acuity benefit) of the amblyopic eye measured after 2 months of treatment, and establish whether it is correlated with the magnitude of short-term monocular deprivation–induced visual plasticity before treatment. Therefore, we plotted the change in perceptual eye dominance from short-term deprivation against the change in acuity from classical patching, as this is the only valid way of assessing whether changes in short-term plasticity can predict improvements in long-term patching. According to Amblyopia PPP (Wallace et al., 2018), the success of patching therapy only makes sense if it is measured in terms of an incremental change (i.e., “improvement”). After replotting Lunghi et al. (2016) data in the more conventional way (corresponding to our data Figure 3A), where perceptual eye dominance changes are plotted against patching-induced acuity changes, their results agree with ours and show no significant correlation (Lunghi et al: *r* = −0.22, *P* = 0.54; the present study: *r* = 0.20, *P* = 0.66) between perceptual eye dominance changes at the beginning of patching therapy and acuity changes after 2 months of classical patching therapy.

There are a number of important differences between our study and the previous one by Lunghi et al. (2016). However, because the data from the two studies are consistent in that there is no significant correlation between short-term perceptual eye dominance plasticity changes and longer-term patching therapy improvements, none of these differences can be particularly crucial. Thus, despite the differences, our conclusions are well founded. Our conclusions are outlined below:

First, Lunghi et al. (2016) used binocular rivalry to measure perceptual eye dominance, whereas we used a binocular combination task. Both tasks are laboratory-based tests and are potentially challenging for children to accurately complete. In the study of Lunghi et al. (2016), they added features to make the test child-friendly. In our study, we included practice sessions and chose patients who were able to do the test accurately. This form of accuracy was verified by looking at the *R*^2^ values for the “binocularly perceived phase versus interocular contrast ratio” curve; if they were larger than 0.85, then the children performed well to be eligible for the study. Nevertheless, both tests have been widely used in studying the homeostatic plasticity in normal adults (rivalry: Lunghi et al., 2011, 2013, 2015b; Lunghi and Sale, 2015; Bai et al., 2017; Kim et al., 2017; Ramamurthy and Blaser, 2018; Finn et al., 2019; Sheynin et al., 2019a, b; combination: Zhou et al., 2013a, 2014, 2017a,b; Bai et al., 2017; Wang et al., 2017; Yao et al., 2017; Min et al., 2018, 2019; Sheynin et al., 2019a) and in patients with amblyopia (rivalry: Lunghi et al., 2019; combination: Zhou et al., 2013c, 2019).

Second, Lunghi et al. (2016) measured perceptual eye dominance associated with short-term occlusion of the fellow fixing eye, whereas we measured changes in perceptual eye dominance associated with short-term deprivation of the amblyopic eye. To our limited knowledge, it is so far not clear whether the effect of short-term patching differs in magnitude for patching different eyes. We have no reason to believe that the underlying mechanisms in patching different eyes are different. We are at present assuming this. In support of this assumption, both studies found similar directions of perceptual eye dominance shift (in favor of the patched eye) in most of the patients (9/10 in Lunghi and colleagues’ study and 5/7 in the present study) after the short-term monocular deprivation. Limited by the small sample in the present study, the normalized perceptual eye dominance indices for the seven patients were not statistically larger than 0 [*t*(6) = 1.33, *P* = 0.23]. However, we still failed to find any significantly correlation between the amblyopic eye acuity improvement after 2 months of occlusion treatment and the normalized perceptual eye dominance index difference associated with the perceptual eye dominance plasticity based on those having an perceptual eye dominance shift in favor of the patched eye (*n* = 5, *r* = −0.14, *P* = 0.82).

In addition, their patients had mild–moderate amblyopia (≤0.4 logMAR) and were treated by occluding the fellow eye with a Bangerter filter (strength 0.4), whereas our patients had more severe amblyopic >0.45 logMAR and were treated by occluding the fellow eye with an opaque patch.

Furthermore, the main conclusions in both the Lunghi et al. (2016) and the current study relied on correlations in small samples [i.e., 7 in ours and 10 in Lunghi et al. (2016)]. It is always hard to justify what is the proper sample size for a valid conclusion based on a correlation analysis; for example, why 10 is enough, whereas 7 is not acceptable? This itself is tightly linked to the question that one asks. In particular, for the question that we asked, whether the homeostatic plasticity predicts the recovery rate (or the effects of occlusion therapy, or the visual acuity improvement), both Lunghi and colleagues’ and our study failed to reach a significant correlation. Thus, both studies suggest that the homeostatic plasticity might not be able to predict the acuity improvements from occlusion therapy. Considering that this conclusion relies on two studies (Lunghi and colleagues’ and ours) with 17 patients (10 in Lunghi and colleagues’ and 7 in ours) from two independent groups using different techniques, we believe that this strengthens the conclusion. In other words, if one must get a large sample to reach significance in this kind of correlation analysis, it is hard to believe that we can use the homeostatic plasticity as a prediction index in clinical practice.

Moreover, in Lunghi and colleagues’ study, compliance was monitored via parents’ reports, whereas in our study no compliance measure was used. Because data from the two studies come to the same conclusion, the fact that compliance was not monitored in the current study cannot be critical to our conclusions.

In summary, we aimed to investigate whether the short-term patching-induced perceptual eye dominance shift *per se* is a good indicator of the visual acuity improvement after patching therapy. We show that short-term perceptual eye dominance plasticity does not provide an index of cortical plasticity in the general sense, such that it could be used to predict acuity improvement outcomes from classical patching. This conclusion is robust to the type of measurement method used, the degree of amblyopia treated, the eye that is occluded (i.e., fixing vs. amblyopic) in the short-term perceptual eye measurement, and the extent to which compliance is monitored.

It remains a possibility that the variability in monocular visual outcome (e.g., visual acuity) following monocular patching therapy could derive not from a difference in plasticity capacity but from a variety of other factors including the amblyopia phenotype (Tacagni et al., 2007), treatment compliance (Beardsell et al., 1999; Menon et al., 2005; Wallace et al., 2018; Handa and Chia, 2019), lifestyle or environment (Woodruff et al., 1994), level of activity, or a plethora of other factors (Stewart et al., 2005; Awan et al., 2010) that do not relate to the inherent capacity for plasticity.

## Data Availability Statement

The datasets generated for this study are available on request to the corresponding author.

## Ethics Statement

The studies involving human participants were reviewed and approved by the Ethics Committee of Wenzhou Medical University. Written informed consent to participate in this study was provided by the participants’ legal guardian/next of kin.

## Author Contributions

CT, JZ, and RH conceived the experiments. CT, ZH, and YC performed the experiments. CT, ZH, YC, and JZ analyzed and interpreted the data. CT, JZ, and RH wrote the manuscript. All authors contributed to the manuscript revision, read, and approved the submitted version.

## Conflict of Interest

The authors declare that the research was conducted in the absence of any commercial or financial relationships that could be construed as a potential conflict of interest.

## References

[B1] Awan M. Proudlock F. A. Grosvenor D. Choudhuri I. Sarvanananthan N. Gottlob I. (2010). An audit of the outcome of amblyopia treatment: a retrospective analysis of 322 children. *Br. J. Ophthalmol.* 94 1007–1011. 10.1136/bjo.2008.154674 19955200

[B2] Bai J. Dong X. He S. Bao M. (2017). Monocular deprivation of Fourier phase information boosts the deprived eye’s dominance during interocular competition but not interocular phase combination. *Neuroscience* 352 122–130. 10.1016/j.neuroscience.2017.03.053 28391010

[B3] Beardsell R. Clarke S. Hill M. (1999). Outcome of occlusion treatment for amblyopia. *J. Pediatr. Ophthalmol. Strabismus.* 36 19–24. 10.1097/00043426-199901000-199901024 9972510

[B4] Binda P. Kurzawski J. W. Lunghi C. Biagi L. Tosetti M. Morrone M. C. (2018). Response to short-term deprivation of the human adult visual cortex measured with 7T BOLD. *eLife* 7:e40014. 10.7554/eLife.40014 30475210 PMC6298775

[B5] Brainard D. H. (1997). The psychophysics toolbox. *Spat Vis.* 10 433–436. 10.1163/156856897x00357 9176952

[B6] Chadnova E. Reynaud A. Clavagnier S. Hess R. F. (2017). Short-term monocular occlusion produces changes in ocular dominance by a reciprocal modulation of interocular inhibition. *Sci. Rep.* 7:41747. 10.1038/srep41747 28150723 PMC5288724

[B7] Ding J. Sperling G. (2006). A gain-control theory of binocular combination. *Proc. Natl. Acad. Sci. U.S.A.* 103 1141–1146. 10.1073/pnas.0509629103 16410354 PMC1347993

[B8] Finn A. E. Baldwin A. S. Reynaud A. Hess R. F. (2019). Visual plasticity and exercise revisited: no evidence for a “cycling lane”. *J. Vis.* 19:21. 10.1167/19.6.21 31246227

[B9] Fronius M. Cirina L. Ackermann H. Kohnen T. Diehl C. M. (2014). Efficiency of electronically monitored amblyopia treatment between 5 and 16 years of age: new insight into declining susceptibility of the visual system. *Vis. Res.* 103 11–19. 10.1016/j.visres.2014.07.018 25130409

[B10] Handa S. Chia A. (2019). Amblyopia therapy in Asian children: factors affecting visual outcome and parents’ perception of children’s attitudes towards amblyopia treatment. *Singapore Med. J.* 60 291–297. 10.11622/smedj.2018151 30488078 PMC6595064

[B11] Hess R. F. Thompson B. (2015). Amblyopia and the binocular approach to its therapy. *Vision Res.* 114 4–16. 10.1016/j.visres.2015.02.009 25906685

[B12] Huang C. B. Zhou Y. Lu Z. L. (2008). Broad bandwidth of perceptual learning in the visual system of adults with anisometropic amblyopia. *Proc. Natl. Acad. Sci. U.S.A.* 105 4068–4073. 10.1073/pnas.0800824105 18316716 PMC2268837

[B13] Kim H. W. Kim C. Y. Blake R. (2017). Monocular perceptual deprivation from interocular suppression temporarily imbalances ocular dominance. *Curr. Biol.* 27 884–889. 10.1016/j.cub.2017.01.063 28262490

[B14] Lunghi C. Berchicci M. Morrone M. C. Di Russo F. (2015a). Short-term monocular deprivation alters early components of visual evoked potentials. *J. Physiol.* 593 4361–4372. 10.1113/jp270950 26119530 PMC4594246

[B15] Lunghi C. Emir U. E. Morrone M. C. Bridge H. (2015b). Short-term monocular deprivation alters GABA in the adult human visual cortex. *Curr. Biol.* 25 1496–1501. 10.1016/j.cub.2015.04.021 26004760 PMC5040500

[B16] Lunghi C. Burr D. C. Morrone C. (2011). Brief periods of monocular deprivation disrupt ocular balance in human adult visual cortex. *Curr. Biol.* 21 R538–R539. 10.1016/j.cub.2011.06.004 21783029

[B17] Lunghi C. Burr D. C. Morrone M. C. (2013). Long-term effects of monocular deprivation revealed with binocular rivalry gratings modulated in luminance and in color. *J. Vis.* 13:1. 10.1167/13.6.1 23637272

[B18] Lunghi C. Morrone M. C. Secci J. Caputo R. (2016). Binocular rivalry measured 2 hours after occlusion therapy predicts the recovery rate of the Amblyopic Eye in Anisometropic children. *Invest. Ophthalmol. Vis. Sci.* 57 1537–1546. 10.1167/iovs.15-18419 27046118 PMC4909145

[B19] Lunghi C. Sale A. (2015). A cycling lane for brain rewiring. *Curr. Biol.* 25 R1122–R1123. 10.1016/j.cub.2015.10.026 26654367 PMC5040496

[B20] Lunghi C. Sframeli A. T. Lepri A. Lepri M. Lisi D. Sale A. (2019). A new counterintuitive training for adult amblyopia. *Ann. Clin. Transl. Neurol.* 6 274–284. 10.1002/acn3.698 30847360 PMC6389748

[B21] Menon V. Chaudhuri Z. Saxena R. Gill K. Sachdeva M. M. (2005). Factors influencing visual rehabilitation after occlusion therapy in unilateral amblyopia in children. *Indian J. Med. Res.* 122 497–505. 10.1089/hum.2005.16.1484 16518000

[B22] Min S. H. Baldwin A. S. Hess R. F. (2019). Ocular dominance plasticity: a binocular combination task finds no cumulative effect with repeated patching. *Vis. Res.* 161 36–42. 10.1016/j.visres.2019.05.007 31194984

[B23] Min S. H. Baldwin A. S. Reynaud A. Hess R. F. (2018). The shift in ocular dominance from short-term monocular deprivation exhibits no dependence on duration of deprivation. *Sci. Rep.* 8:17083 10.1038/s41598-018-35084-35081 PMC6244356 30459412

[B24] Mou T. (1966). Logarithmic visual acuity chart and five-score recording. *Chin. J. Ophthalmol.* 13 96–106.

[B25] Pelli D. G. (1997). The VideoToolbox software for visual psychophysics: transforming numbers into movies. *Spat Vis.* 10 437–442. 10.1163/156856897x00366 9176953

[B26] Ramamurthy M. Blaser E. (2018). Assessing the kaleidoscope of monocular deprivation effects. *J. Vis.* 18:14. 10.1167/18.13.14 30572342

[B27] Sheynin Y. Chamoun M. Baldwin A. S. Rosa-Neto P. Hess R. F. Vaucher E. (2019a). Cholinergic potentiation alters perceptual eye dominance plasticity induced by a few hours of monocular patching in adults. *Front. Neurosci.* 13:22. 10.3389/fnins.2019.00022 30766471 PMC6365463

[B28] Sheynin Y. Proulx S. Hess R. F. (2019b). Temporary monocular occlusion facilitates binocular fusion during rivalry. *J. Vis.* 19:23. 10.1167/19.5.23 31136647

[B29] Stewart C. E. Fielder A. R. Stephens D. A. Moseley M. J. (2005). Treatment of unilateral amblyopia: factors influencing visual outcome. *Invest. Ophthalmol. Vis. Sci.* 46 3152–3160. 10.1167/iovs.05-0357 16123414

[B30] Stewart C. E. Stephens D. A. Fielder A. R. Moseley M. J. (2007). Objectively monitored patching regimens for treatment of amblyopia: randomised trial. *BMJ* 335:707. 10.1136/bmj.39301.460150.55 17855283 PMC2001048

[B31] Tacagni D. J. Stewart C. E. Moseley M. J. Fielder A. R. (2007). Factors affecting the stability of visual function following cessation of occlusion therapy for amblyopia. *Graefes Arch. Clin. Exp. Ophthalmol.* 245 811–816. 10.1007/s00417-006-0395-392 17047980

[B32] Wallace D. K. Repka M. X. Lee K. A. Melia M. Christiansen S. P. Morse C. L. (2018). Amblyopia preferred practice pattern(R). *Ophthalmology* 125 105–142. 10.1016/j.ophtha.2017.10.008 29108744

[B33] Wang Y. Yao Z. He Z. Zhou J. Hess R. F. (2017). The cortical mechanisms underlying ocular dominance plasticity in adults are not orientationally selective. *Neuroscience* 367 121–126. 10.1016/j.neuroscience.2017.10.030 29111362

[B34] Woodruff G. Hiscox F. Thompson J. R. Smith L. K. (1994). Factors affecting the outcome of children treated for amblyopia. *Eye* 8(Pt 6), 627–631. 10.1038/eye.1994.157 7867817

[B35] Yao Z. He Z. Wang Y. Lu F. Qu J. Zhou J. (2017). Absolute not relative interocular luminance modulates sensory eye dominance plasticity in adults. *Neuroscience* 367 127–133. 10.1016/j.neuroscience.2017.10.029 29111363

[B36] Zhou J. Baker D. H. Simard M. Saint-Amour D. Hess R. F. (2015). Short-term monocular patching boosts the patched eye’s response in visual cortex. *Restor. Neurol. Neurosci.* 33 381–387. 10.3233/rnn-140472 26410580 PMC4923712

[B37] Zhou J. Clavagnier S. Hess R. F. (2013a). Short-term monocular deprivation strengthens the patched eye’s contribution to binocular combination. *J. Vis.* 13:12. 10.1167/13.5.12 23599416

[B38] Zhou J. Huang P. C. Hess R. F. (2013b). Interocular suppression in amblyopia for global orientation processing. *J. Vis.* 13:19. 10.1167/13.5.19 23608341

[B39] Zhou J. Thompson B. Hess R. F. (2013c). A new form of rapid binocular plasticity in adult with amblyopia. *Sci. Rep.* 3:2638. 10.1038/srep02638 24026421 PMC3770967

[B40] Zhou J. He Z. Wu Y. Chen Y. Chen X. Liang Y. (2019). Inverse occlusion: a binocularly motivated treatment for Amblyopia. *Neural Plast.* 2019:5157628. 10.1155/2019/5157628 31015829 PMC6444262

[B41] Zhou J. Reynaud A. Hess R. F. (2014). Real-time modulation of perceptual eye dominance in humans. *Proc. Biol. Sci.* 281:20141717. 10.1098/rspb.2014.1717 25274364 PMC4213622

[B42] Zhou J. Reynaud A. Hess R. F. (2017a). Aerobic exercise effects on ocular dominance plasticity with a phase combination task in human adults. *Neural Plast.* 2017:4780876. 10.1155/2017/4780876 28357142 PMC5357532

[B43] Zhou J. Reynaud A. Kim Y. J. Mullen K. T. Hess R. F. (2017b). Chromatic and achromatic monocular deprivation produce separable changes of eye dominance in adults. *Proc. Biol. Sci.* 284:20171669. 10.1098/rspb.2017.1669 29142113 PMC5719170

[B44] Zhou Y. Huang C. Xu P. Tao L. Qiu Z. Li X. (2006). Perceptual learning improves contrast sensitivity and visual acuity in adults with anisometropic amblyopia. *Vis. Res.* 46 739–750. 10.1016/j.visres.2005.07.031 16153674

